# Enigmatic Headstands in European Freshwater Fish Species

**DOI:** 10.1002/ece3.71005

**Published:** 2025-02-15

**Authors:** Michal Tušer, Jaroslava Frouzová

**Affiliations:** ^1^ Institute of Hydrobiology Biology Centre of the Czech Academy of Sciences České Budějovice Czech Republic

**Keywords:** body posture, communicative signal, European fish species, fish behavior, head‐down display, tail‐up

## Abstract

This study discloses a remarkable and rarely documented behavior among two cyprinid species—the common bleak ( *Alburnus alburnus* ) and the common roach ( *Rutilus rutilus* )—and one percid species, the European perch ( *Perca fluviatilis* ). The primary aim was to reevaluate this distinct behavior, termed “headstand”, and explore its potential implications for fish behavior and ecology. We utilized video recording to observe and analyze this behavior. Fish were monitored in an enclosure with near‐natural conditions. Instances of headstanding behavior—where fish assume a head‐down posture at angles between 45° and 90°—were documented. The video analysis revealed the occurrence of headstanding behavior in all three species, lasting from a few seconds to over a minute. This behavior was observed both individually and in groups, sometimes involving multiple species simultaneously. Group headstands occurred more frequently in the year when water transparency was low. Interestingly, although this behavior was previously proposed as a cleaning signal, no cleaning activity was observed afterward. Our observations suggest that headstanding may serve additional functions beyond a cleaning context, potentially involving foraging, predator avoidance, or social communication. Observing this behavior under diverse conditions highlights its ecological and social relevance. Future work integrating behavioral, ecological, and physiological approaches could clarify the adaptive significance of headstanding employed by these species.

## Graphical abstract

This study documents a distinct and rarely documented behavior, termed “headstand”, in juvenile common bleak (*Alburnus alburnus*), common roach (*Rutilus rutilus*), and European perch (*Perca fluviatilis*). Observed through video recordings, headstanding—where fish assume a head‐down posture—appears linked to social interaction, environmental adaptation, and survival strategies.

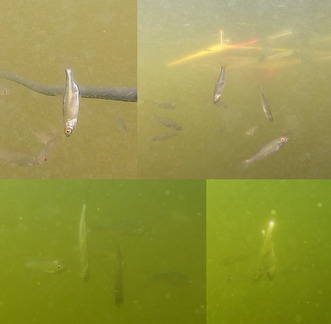

## 1 Introduction

**FIGURE 1:**
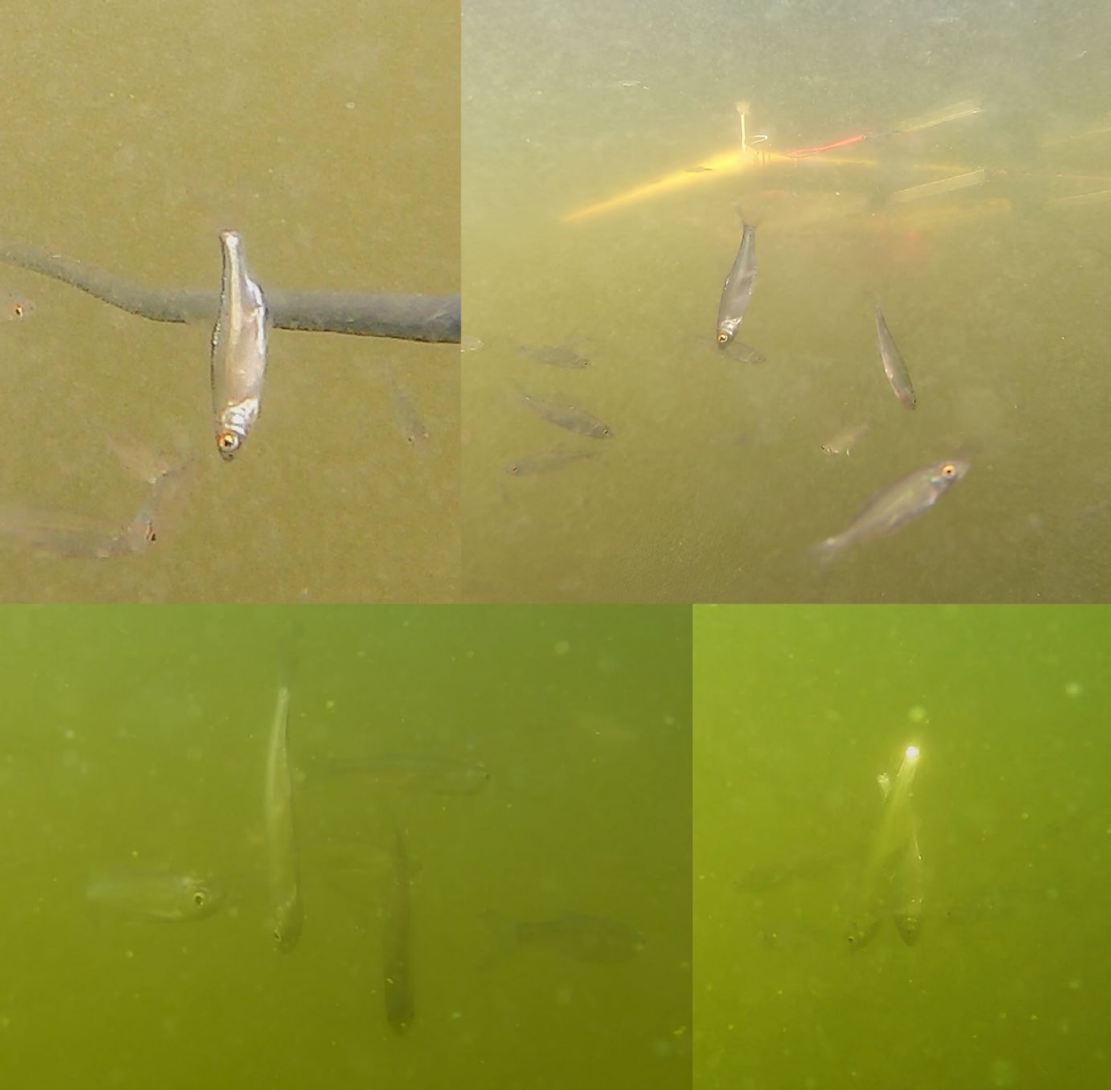
The headstanding juveniles of the common roach, *Rutilus rutilus* (upper images), and the common bleak, *Alburnus alburnus* (lower images), recorded in the Czech Republic in 2019 and 2022, respectively, are surrounded by their swimming conspecifics.

During an incidental observation while conducting another scientific study on the open water in the middle of Europe, we witnessed headstanding behavior in juvenile roach ( *Rutilus rutilus* ) and bleak ( *Alburnus alburnus* ), occasionally with perch ( *Perca fluviatilis* ) (Figure 1). Not only individually, but these fish formed headstanding groups that even included a diverse mix of the given species—a rarely documented aspect of their behavioral repertoire.

Although Abel (1971) described headstanding in these species, our observations suggest that this behavior may serve purposes beyond those previously considered. This headstanding resembles displays noted in tropical fish and even parallels Abel's (1971) observations in the standing waters of the Danube river, where it was linked to cleaning interactions. Abel's explanation, along with the idea that our understanding of the behavioral ecology of these common freshwater species may be incomplete, piqued our scientific curiosity and prompted further exploration.

Fish body orientation plays a crucial role, finely tuned to the specific ecological niches and environmental conditions fish inhabit. From streamlined swimmers to vertical pioneers like the shrimpfish (subfamily Centriscinae), fish exhibit a wide spectrum of distinctive body orientations that enable them to thrive in various aquatic environments. While the natural and anticipated body orientation is horizontal, with swimming facing forward and effectively moving from one point to another, the emergence of alternative orientations—particularly vertical ones where the head is directed either downward or upward—introduces intriguing possibilities for niche applications or alternative uses. Adopting a vertical orientation, as seen in some species, can offer unique advantages, albeit at a potentially significant energetic cost.

In the dynamic aquatic environment, the pursuit of vertical posing is a multifaceted challenge that is closely intertwined with the behavior of fish species worldwide. While some advanced teleosts, exemplified by the enigmatic shrimpfish ( *Aeoliscus punctulatus* ), have honed specialized adaptations enabling them to navigate with remarkable precision in a head‐down position (Fish and Holzman 2019), the broader spectrum of fish species employs vertical body orientation as a transient yet vital posture. The fish's vertical orientation serves various purposes essential to their survival, including evading predators (Brandl and Bellwood 2015; Foam et al. 2005; Kondo and Abe 1995; Miller et al. 2014), foraging (Auster 2010; Keenleyside 1955; Lauridsen et al. 2022; Miller et al. 2014), and communication within their complex interactions involving reproductive dynamics (Baynes et al. 1994; Ladich 2007; Mclennan 2005; Rowland et al. 2002; Victor 1987), social hierarchies (Manara et al. 2022; Ruberto et al. 2020), and cleaning behaviors (Able 1976; Minahull 1985; Shepherd et al. 2005).

Expanding upon these ecological functions, examples of vertical posturing in fish encompass a wide range of behaviors tailored to specific ecological contexts. In terms of antipredatory tactics, bigeye lates ( *Lates mariae* , Cetropomidae) exhibit effective head‐down camouflaging techniques (Kondo and Abe 1995), rabbitfishes (family Siganidae) are known for their vigilant head‐up guarding behaviors (Brandl and Bellwood 2015), and starry rays employ a defensive head‐up posture when confronted with threats (Martin and Rekdal 2006). Foraging strategies among these organisms are equally diverse. Predator hunting behaviors are exemplified by lionfish, combining head‐down and head‐up posturing (García‐Rivas et al. 2018), and head‐up snipe eels (Miller et al. 2014), whereas others, like the coelacanth, engage in head‐down substrate probing to secure prey (Lauridsen et al. 2022). Communication within these species is intricate and multifaceted. Courtship rituals using the head‐down posture are observed in species, such as the topmouth gudgeon *Pseudorasbora parva* (Burnard et al. 2012), swordtails *Xiphophorus cortezi* (Fernandez et al. 2008), *X. nezahualcoyotl* (Lyons and Morris 2008), and smallmouth bass *Micropterus dolomieui* (Ridgway et al. 1989). Social dynamics include various displays such as head‐down threat displays seen in cichlids *Haplochromis burtoni* (Heiligenberg et al. 1972) and *Lamprologus ocellatus* (Walter and Trillmich 1994), as well as dominance assertions and submission cues demonstrated, for instance, by the head‐up posturing daffodil cichlid *Neolamprologus pulcher* (Manara et al. 2022; Ruberto et al. 2020). Additionally, cleaning behaviors, indicative of mutualistic interactions between species, are observed using head‐down or head‐up posturing signals, as seen in the example of cichlid *Tilapia rendalli* (Minahull 1985).

Despite the prevalence of vertical posing among tropical and marine fish, European freshwater ecosystems host only a handful of native species known for such behavior. Among these, vertical orientation plays diverse roles. The well‐studied stickleback is renowned for the head‐up posture in females indicating mating readiness (Rowland et al. 2002). The northern pike ( *Esox lucius* ) employs intraspecific antipredatory head‐up displays (Peukert 2011), and the sunbleak ( *Leucaspius delineatus* ) showcases a distinctive female head‐down posture during courtship (Gozlan et al. 2003). Head‐down postures, slightly inclined in bitterling ( *Rhodeus amarus* ) and perch and strongly inclined in bleak, roach, and rudd ( *Scardinius erythrophthalmus* ), were also observed and interpreted as a cleaning signal (Abel 1971). Understanding the nuances of vertical orientation in fish offers profound insights into their ecology, behavior, and evolutionary adaptations, particularly when considering the energetic costs and benefits of such behaviors.

Building on this knowledge, our study revisits the head‐down posture in roach, perch, and bleak, aiming to reevaluate its ecological and behavioral roles. Specifically, we document and characterize the headstanding behavior in juvenile fish of these three common European species, emphasizing its ecological and behavioral significance. While Abel (1971) relied on direct visual observations by divers to describe this behavior, our study utilizes extensive video analysis, allowing for more detailed and systematic assessments. We provide a comprehensive description of the behavior and assess its occurrence, duration, and interindividual variation. In addition, we discuss plausible causes underlying this behavior, considering physiological, ecological, and social factors. By placing these findings in a broader behavioral ecological framework, we aim to advance the understanding of fish behavior and its adaptive functions across species.

## 2 Materials and Methods

### 2.1 Study Site

The observations for this study originate from the dam area of the Římov Reservoir (48°50′ N, 14°28′ E) during the summer and fall of both 2019 and 2022. The reservoir is identified as a meso‐eutrophic water body. Phytoplankton concentrations in the reservoir dam area were approximately 16 and 49 μg/L of Chlorophyll α (ethanol) in 2019 and 2022, respectively, the latter being significantly higher (Table S1, Figure S1).

Video recordings of headstanding behavior in fry were obtained only within an enclosure that allowed detailed behavioral observations, which would otherwise be challenging in nonconfined open waters with naturally low fish concentrations. The enclosure consisted of a circular float with an inner diameter of 5 m, complemented by an 8‐m deep cylindrical net with a mesh size of 10 mm. The enclosure was placed in the pelagic zone of the lake and attracted fry, resulting in a higher concentration of fish in a smaller area of the pelagic zone. During the respective seasons, fry were able to enter and leave the enclosure freely through the net's meshes, interacting with the enclosure in a similar way to natural habitat structures, such as plants or submerged wood, which they use as refuges or spatial markers in open waters (Braithwaite 1998; Castro et al. 2002).

The fry population consisted of roach, bleak, bream ( *Abramis brama* ), and perch with a total length ranging from 27 to 70 mm and averaging 51 mm. The enclosure also housed either three adult asp (20 cm) or two adult perch (26 cm) in 2019 and two adult asp (20 cm) together with up to eight adult bleaks in 2022.

The headstanding behavior of fry and adult fish seen outside of the enclosure was not videotaped and is only discussed in this article.

### 2.2 Video Recording and Processing

In this investigation, video data acquisition was conducted through the utilization of multiple GoPro HERO7 Black cameras (GoPro Inc., San Mateo, California, USA). Within the field setting, these cameras were deployed in various configurations to capture specific observational ranges, including fixed positions directed toward the center or along the inner periphery of the enclosure, as well as hand‐held, affording adaptable maneuverability. The stationary cameras were precisely situated several centimeters beneath the water's surface, with their lenses oriented either horizontally or at a slight upward inclination. Center‐aimed cameras were fixed 30–40 cm inward from the inner enclosure periphery, directed toward the center to cover the entire area from this point to the center. In contrast, cameras aimed along the inner periphery observed activity within a range extending from the periphery itself to 30–40 cm inward.

Following the completion of data collection, a systematic manual processing of all recorded videos was undertaken. This postcollection processing was facilitated using the freely available GOM player (GOM & Company, Seoul, Republic of Korea; version 2.3.89).

When analyzing the recorded videos, we identified and classified different behaviors of the fish in terms of movement and body orientation. Swimming, characterized by active, directed movements, constituted one category. The other category, nonswimming (hovering), encompassed behaviors where the fish maintained a stationary position. Within the nonswimming category, we further subdivided based on body orientation.

**FIGURE 2:**
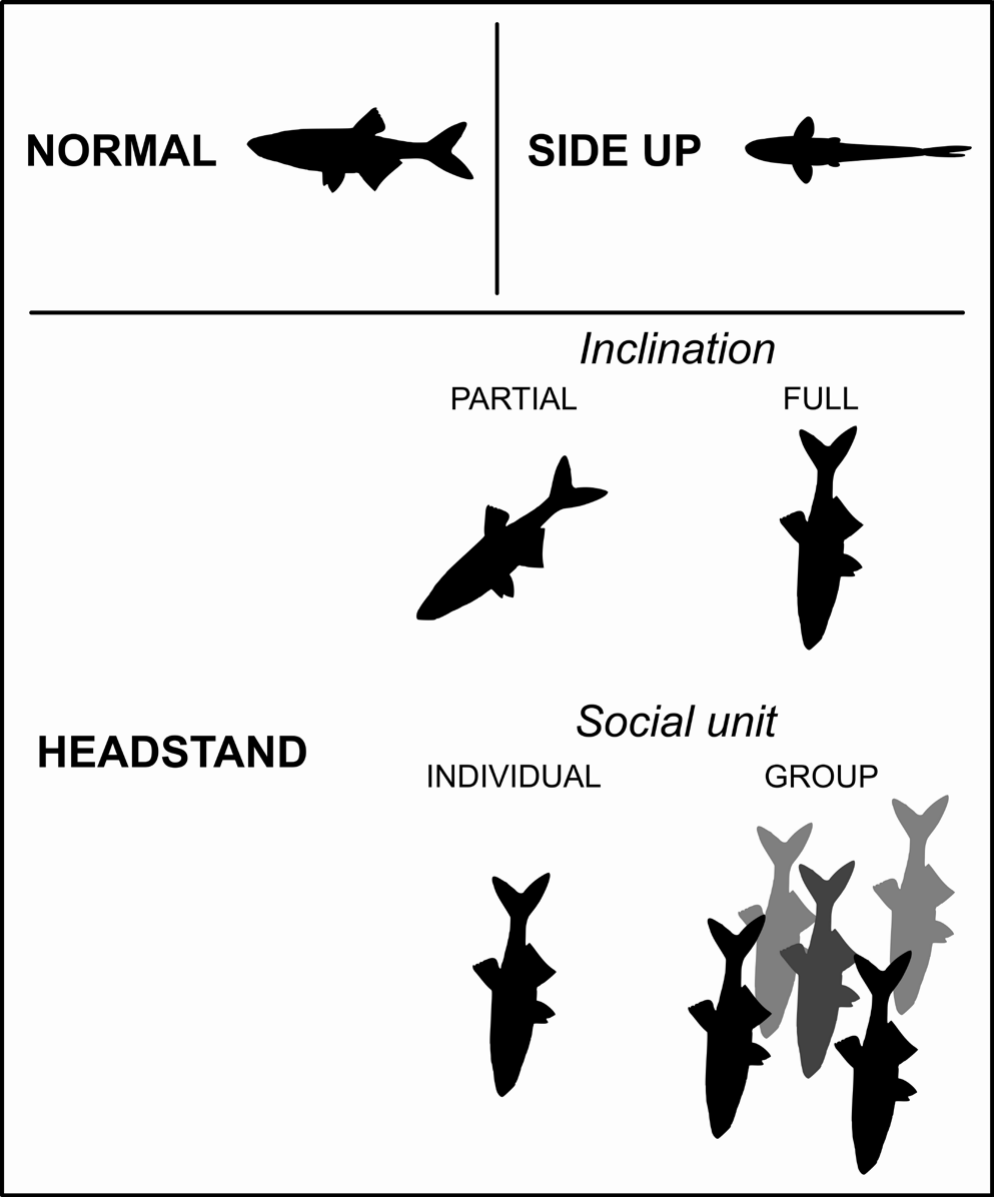
Schematic side‐view representation of nonswimming behaviors based on fish body orientation.

Nonswimming behaviors included (Figure 2):

1. Normal orientation
2. Side‐up orientation: This refers to a fish maintaining a stationary position with its side directed upward (not included in this study).
3. Head‐down orientation (referred to as “headstand”): In this behavior, a fish maintains a stationary position with its head directed downward. The headstanding behavior is subdivided into
  - Based on inclination
    - Partial headstand (~45°)
    - Full headstand (90°)
  - Based on social units
    - Individual headstand
    - Group headstand: Denoting instances where two or more fish are in close proximity (maximum a few centimeters apart) engaging in the headstanding behavior, encompassing partial, full, or a combination thereof.

In the records, attention was drawn exclusively to two nonswimming behaviors, namely head‐down and side‐up orientations. However, the side‐up orientation was excluded from the scope of this study. When a headstand was evident, it was categorized as partial, full, or a combination of both to account for the possibility that a single fish may change the angle of the headstand display within the event. Unfortunately, it was not always possible to accurately determine the angle of the body. The body angle was not processed further. Each event involving these behaviors was marked at its onset and conclusion. The fish involved in each event were quantified and, where possible, identified to species level. For each observed event, the duration was calculated to capture the range of behaviors exhibited. In cases where a collective manifestation of the behavior occurred within a group of fish, the total duration represented the aggregated time that the behavior was displayed collectively, rather than separating individual durations within the group.

## 3 Results

Over a cumulative duration exceeding 14 h of video recordings in two different time periods (2.5 h in 2019 and 12 h in 2022), a total of 115 events were documented in which over 215 fish performed a headstand either individually or in a group (Table 1). Within the enclosure, all headstands were performed in the wider central area, with none occurring near the enclosure walls (no headstand recorded by the cameras directed along the inner periphery). Additionally, the headstands were mainly observed within the first half meter below the water surface due to the water transparency and the coverage by the cameras.

**TABLE 1:** A quantitative analysis of the duration of headstanding behavior performances.

Year	Number of individuals	Number of events^a^	Headstand duration (s)			
			Mean	SD	Min	Max
2019	1	67	9.2	10.2	0.4	59.2
	2	6	12.2	7.2	3.8	22.1
	3	2	10.5	5.2	6.8	14.2
	4	3	22.0	15.2	8.5	38.5
	≥ 5					
	Total	78	9.9	10.2	0.4	59.2
2022	1	13	6.0	6.4	0.9	25.4
	2	5	16.4	9.5	4.0	27.8
	3	4	18.8	15.9	7.7	42.2
	4	3	18.6	13.3	9.7	34.0
	≥ 5	12	31.9	22.2	6.9	80.3
	Total	37	18.2	18.0	0.9	80.3
Both	1	80	8.7	9.7	0.4	59.2
	2	11	14.1	8.2	3.8	27.8
	3	6	16.0	13.2	6.8	42.2
	4	6	20.3	12.9	8.5	38.5
	≥ 5	12	31.9	22.2	6.9	80.3
	Total	115	12.6	13.7	0.4	80.3

Abbreviation: SD, Standard deviation.

^a^ The number of events that occurred with a given number of headstanding individuals.

Due to the suboptimal water conditions and the small size of the fish, it was difficult to fully describe the mechanism of the headstands and capture all the nuances of the fin movements, which were barely visible. It appears that the fish had no difficulty achieving the headstand, whether from a stationary position or from movement. The movement was smooth. Simply, the main motor was likely the pectoral fins with stronger rear beating and light beating of the lower part of the tail. The flapping of the pectoral fins was also responsible for maintaining the pose. Some corrections to the pose were made by the tail and the pelvic fins. To exit the pose, a fast and powerful tail stroke was usually performed. The body angle of the headstand pose varied from partial to full, approximately 45°–90°. Occasionally, the pose was accompanied by tail or body quivering (Ruberto et al. 2020).

**FIGURE 3:**
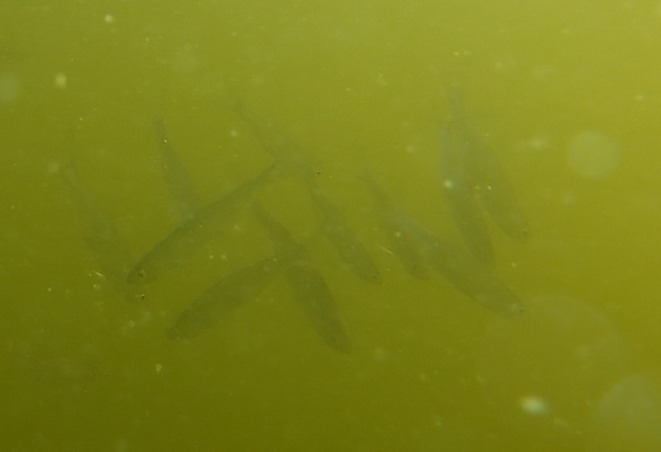
The group of the common bleak ( *Alburnus alburnus* ) performs a collective partial headstand.

In 2019, we recorded approximately 100 individuals performing the headstand mostly individually, whereas in 2022, we observed over 120 individuals more frequently in the group headstand (Figure 3). Notably, the water transparency was much worse in 2022 compared to 2019, suggesting that decreased visibility may have influenced the increase in group headstands. The headstands lasted between half a second and over a minute. The more individuals involved in the group headstand, the longer the headstand lasted on average (Table 1). The group headstands with over five individuals were the longest (80 s in the recording).

**FIGURE 4:**
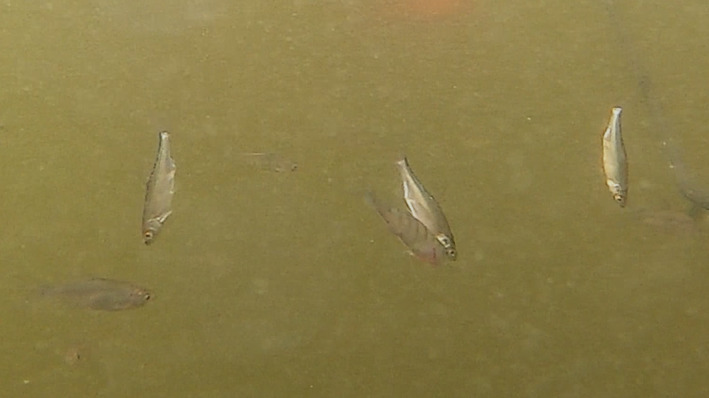
The perch ( *Perca fluviatilis* ) performed the partial headstand with other headstanding roaches ( *Rutilus rutilus* ) in 2019.

**FIGURE 5:**
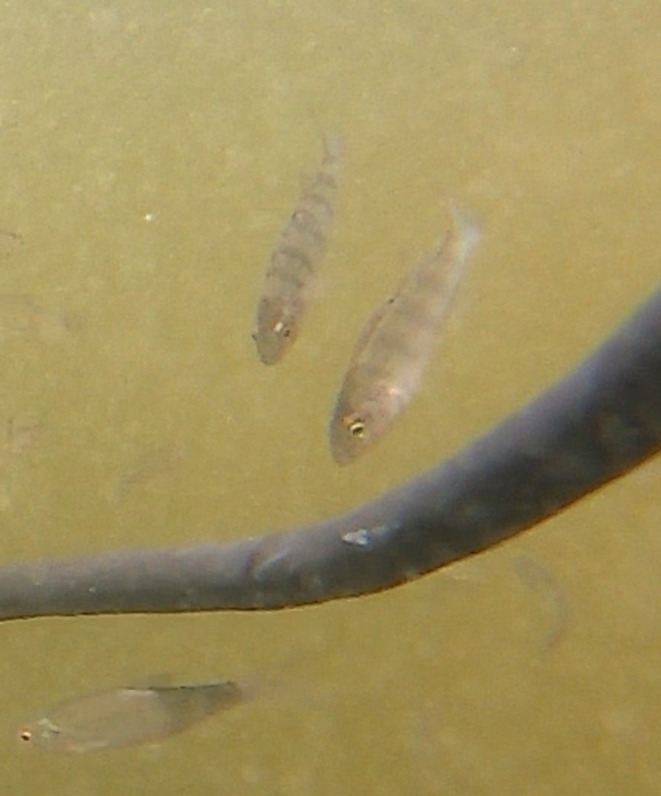
Two headstanding juveniles of the European perch ( *Perca fluviatilis* ) were recorded in 2022 (the Římov Reservoir, Czech Republic). Below is a juvenile roach ( *Rutilus rutilus* ).

The identification of the fish species that showed headstands on the video recordings proved to be difficult due to the poor water quality, in which identification features were not recognizable. On the video recordings, we were able to identify roach (over 50 individuals), bleak (over 25 individuals), and perch (8 individuals). While roach and bleak were visually observed to perform headstands regularly, this behavior was only occasionally visually observed in perch due to a low number in the area. Most of the time, perch only did a partial headstand when approaching other community companions in a headstand (Figure 4). In contrast to visual observations, it was only recorded once with conspecifics (Figure 5). On two occasions in the recordings, it was inconclusive to determine the species, but by the nature of the body, it appeared to be a bream. However, we marked them as unidentified due to the uncertainty and the fact that we did not notice the bream's headstand during our visual observations.

## 4 Discussion

Our research has documented a manifestation of headstanding in common European fish fry, primarily roach and bleak, occasionally observed in perch and with a potential occurrence in a bream. These headstands occurred individually and in groups, with longer durations often observed in group settings, hinting at potential social or environmental influences. Variations in the body angle of the headstand and tail or body quivering in some instances further underscore the complexity of this behavior, suggesting an interplay between individual positioning and group dynamics.

To systematically assess the headstanding witnessed in open water, headstanding behavior was recorded in an enclosure that unintentionally provided an effective environment for detailed observation under near‐natural conditions. The enclosure attracted the fish and lacked physical structures other than the net walls, with no visible bottom, creating an open, unstructured space for these 3–7 cm fish. Despite the presence of the enclosure, the behavior was predominantly observed in the center, away from the walls, suggesting that the fish perceived the central open area to be a more typical environment for this behavior. Without explicit intention to associate this behavior with open waters, we believe that the enclosure did not alter the fish's natural tendencies, making the observations representative and relevant for understanding its occurrence in natural settings.

In attempting to understand the underlying reasons for headstanding behavior, it is crucial to consider the broader behavioral repertoire of fish, especially those belonging to small, highly mobile species. These fish often exhibit continuous swimming behavior, making it difficult to distinguish individuals or discrete behavioral acts (Smith 1991). This impression of uniformity is inconsistent with the behavioral complexity present within these shoaling species and may obscure the intricate social dynamics and adaptive strategies at play. However, their confusing motion and similar physical appearance serve as important elements in their defense against predators. Thus, while headstanding behavior may appear enigmatic, it likely serves a strategic purpose in response to environmental challenges and social interactions within fish communities.

When we first noticed the headstanding behavior, our attention was drawn to the fact that it is widespread beyond the local habitat, extending not only to fry but also to adults, especially adult bleaks. However, we were only able to observe individual, not group, instances of this behavior within adult bleak shoals. Despite our efforts, the vast expanse of open waters, coupled with the low density of the fish, hindered our ability to capture the headstanding behavior among adult bleaks on a record. This limitation highlights the challenges inherent in studying fish behavior, particularly in dynamic and expansive aquatic environments. Notwithstanding these challenges, we managed to obtain a video from a previous project showing an adult roach engaging in headstanding, albeit unfortunately from a considerable distance (Figure S2).

Interestingly, the behavior described above has so far received little attention from scientists. A comprehensive search through scientific databases, such as Web of Science, yielded not much relevant literature on the subject. For the species observed in our study, Abel's study (1971), written in German with an English summary, described behavior in European fish species, including bleak, roach, and perch, that involved a head‐down posture, which he interpreted primarily as cleaning behavior. The only other mention we found for these species was in a popular review of sleep in fish written by Brazzier (2017) for an angling magazine from Ireland. While our results are consistent with the described posture, we observed important differences in behavioral context that suggest alternative interpretations. These differences are explored in detail in the following sections, where we attempt to contribute new perspectives to the understanding of this intriguing behavior.

Starting with the physiological level, the lowest tier in the hierarchy of animal behavior and ecology, internal processes are fundamental to understanding behavior. Brazzier (2017) described similar behavior in juvenile roaches during the day, proposing that the fish may sleep due to a high arousal threshold. However, our observations differed as the fish adopted the same posture while remaining alert. It was difficult to get close to the headstanding fish with the camera. This suggests that the behavior may involve some form of microsleep or relaxation while maintaining awareness (NOAA 2023). If this is the case, the question arises as to why the head‐down posture is physiologically beneficial for sleep or relaxation and why the fish sleep in groups.

Another potential physiological explanation for this behavior is that the headstand may facilitate the expulsion of gas from the swim bladder, particularly in physostomous fish, which possess ducts for venting air. Although Kaartvedt et al. (2021) observed coordinated gas release in pelagic schooling sprats, this phenomenon has not yet been sufficiently explored. No gas bubbles were visible in the observed fish that would have been noticeable in such small objects and with the quality of the recordings available. Additionally, this explanation is further complicated by the presence of headstanding perch as physoclistous fish.

We believe that headstanding is not driven by physiological needs. Moreover, it has been observed under near‐natural conditions and does not appear to be associated with any diseases or physiological abnormalities, such as those caused by food deprivation (Ross et al. 1996). This implies that headstanding may serve a tactical or strategic purpose in response to environmental challenges beyond mere physiology or pathology.

Open waters, where we witnessed the headstanding behavior, pose numerous challenges to freshwater organisms, particularly due to the lack of physical structures for concealment and the constant threat of predator attacks from all directions. Although the behavior for this study was recorded within an enclosure, the conditions inside mimicked these open‐water challenges for these juvenile fish, such as the absence of structural refuges and a featureless environment. This lack of concealment may heighten the importance of group dynamics or specific postures, like headstanding, in mitigating predation risks. For predators in an open‐space environment, launching attacks from dark depths provides a strategic advantage, utilizing their natural countershading to conceal their approach (Donohue et al. 2020; Pembury Smith and Ruxton 2020). While darkness provides effective camouflage, the light falling from the water surface illuminates potential targets in the layers above, making them more visible and easier to target. Given these conditions, performing a headstand underwater can serve as a dual‐purpose tactic: it aids in patrolling the depths while also diminishing a fish's visibility or altering its elongated silhouette when viewed from below or from the side, thereby disguising it as something other than prey. This deceptive posture can confuse predators accustomed to recognizing prey by their typical horizontal orientation and motion (Levin 1997). Furthermore, grouping can enhance the effectiveness of this confusion (Landeau and Terborgh 1986).

When comparing the number of individuals in group headstands in 2019 and 2022, a notable increase was observed in 2022, which coincided with a deterioration in water transparency. This change suggests a possible collective response to adverse environmental changes, with communities adapting their behaviors to mitigate risks such as increasing surveillance to spot predators. By promoting a culture of mutual support and protection, communities may ultimately strengthen their resilience to potential threats and improve their ability to thrive in challenging environments.

The headstanding behavior observed in certain fish species prompts an investigation of its potential relationship to feeding dynamics. In particular, the head‐down posture is advantageous for probing and foraging for bottom‐dwelling prey, such as is performed by coelacanths (Lauridsen et al. 2022) and grenadiers (Auster 2010). Remarkably, this behavior also extends to open waters, exemplified by lionfishes employing it as a primary hunting tactic for small fish (García‐Rivas et al. 2018). The fry of the species investigated in this study have a pronounced focus on foraging for zooplankton. In open waters, the transparency of zooplankton provides an effective strategy to conceal themselves from horizontally scanning eyes, supporting the idea that visual cues are used for prey detection. This stationary headstanding behavior could be consistent with the same principle observed in adult bream and roach, possibly also perch (Martin Čech pers. comm.), which are characterized by mobile, sinusoidal swimming behavior (Čech and Kubečka 2002; Jarolím et al. 2010). The essence is that they visually locate their prey by looking down and using the light scattered in the prey tissue to stand out from darker depths (Thetmeyer and Kils 1995). Furthermore, the phenomenon of headstand grouping remains enigmatic, but one plausible explanation is that it serves as an unintentional signal to indicate the presence of a food patch to passing fish. This signaling function is similar to that observed in three‐spined sticklebacks, where the headstanding behavior attracts conspecifics to areas with abundant food (Keenleyside 1955). However, no increase in feeding activity or pursuit of prey was observed following the headstand, suggesting that this behavior may have other functions.

Given these factors, we propose that headstanding may be a communicative signal rather than a physiological necessity or tactical feature. The body inclination may be correlated with the intensity of expression or motivation for interaction (Able 1976). Additionally, supplementary movements like tail or body quivering could support the signal (Ruberto et al. 2020).

Headstanding is known to be used as a communicative signal between the opposite sexes during courtship rituals in some species, such as smallmouth bass (Ridgway et al. 1989), sunbleak (Gozlan et al. 2003), swordtails (Fernandez et al. 2008; Morris et al. 2008), and topmouth gudgeon (Burnard et al. 2012), but there is currently no documented evidence or confirmed findings indicating its use in the reproductive behavior of the observed species. Furthermore, it seems unlikely that the head‐down posture is directly related to sexual behavior, as observations of headstanding adults have been made outside the typical reproductive periods, and the fry of roach, bleak, and perch are still too young to reproduce. Therefore, the idea that headstanding is a courtship behavior may not be very relevant in this context.

In the absence of evidence supporting a courtship function, an alternative hypothesis is that headstanding is a signal in cleaning‐related behavior, in a system where multiple species understand the same cue (e.g., Able 1976). The cleaning behavior of temperate freshwater fishes was initially interpreted by Abel (1971) and subsequently by Sulak (1975). Abel (1971) proposed that the headstanding behavior served as a signal for cleaning in the species observed in our study, namely bleak, roach, and perch. Nevertheless, he noted that actual instances of cleaning were infrequent. He observed that bleak rarely engaged in cleaning behaviors with other fish, even those of similar size, and that roach frequently stood motionless or headstanding in groups for hours without actively engaging in cleaning. Abel attributed this low cleaning activity to the scarcity of specialized cleaner fish in freshwater habitats and the high prevalence of irritants like fungi alongside plentiful regular food. In other words, the fish desire to be cleaned, but there is no one to do it.

However, our observations suggest a different context. In our visual observations and video recordings, we found no evidence of cleaning (actions like nibbling or rubbing) associated with headstanding at all. This absence raises the possibility that, in these species within open freshwater environments, headstanding may represent a vacuum behavior (Lorenz 1981) or fulfill other ecological or social roles. While headstanding itself may function as a general signaling behavior or postural adjustment, the seldom cleaning events that Abel (1971) observed might instead serve to reinforce social bonds within these groups. This interpretation highlights the versatility of fish postures, suggesting that behaviors traditionally viewed as cleaning may also contribute to social cohesion in freshwater fish communities.

The headstanding behavior observed in both juvenile and adult roach and bleak could serve as a multifaceted communication tool within their social dynamics. Reddon et al. (2021) propose that such transient postural changes can function as submission signals, facilitating the establishment of social order and minimizing conflicts within stable groups of animals. In the case of cyprinids like roach and bleak, which are shoaling prey fish from the larvae, this behavior may signify various intentions beyond mere submission. As juveniles begin to explore their environment and form loose aggregations, the headstanding posture might act as an initiation of communication, soliciting companionship, or signaling a request to a shoal or mate. Indeed, the process of forming cohesive shoals or schools has been linked to benefits such as enhanced foraging success and reduced metabolic rates due to the stress alleviated by group living (Abrahams and Colgan 1985; Nadler et al. 2016; Pitcher et al. 1982). By closely monitoring each other's behavior, fish in these social groups effectively “share” information, with headstanding potentially serving as the first step toward initiating or expanding such cooperative arrangements.

Although headstanding of perch was observed in fewer numbers, and as adults are predatory fish in contrast to roach and bleak, perch is also a shoaling species, and the aforementioned hypothesis above could likely apply to perch as well.

Rather than being limited to a single species, headstanding was observed across multiple fish species in our study, suggesting its potential as a versatile communication tool. Occasionally, headstanding groups composed of various species were observed, suggesting a shared understanding or response to certain ecological or social cues. This finding supports the need to consider headstanding as a multispecies phenomenon rather than a general behavior confined to specific species. While for roach and bleak, with their similar ecology, the signal and purpose may be the same, for perch, it could represent a manifestation of curiosity or other behavioral complexities. This variability in interpretation across species highlights the intricate nature of fish communication and the importance of considering a range of ecological and behavioral factors when studying such phenomena.

At this stage, attempting to pinpoint the primary explanation for the occurrence of headstands is challenging, as the observed headstands may have been executed with varying intentions or a combination thereof. For example, a headstand in a three‐spined stickleback could denote a distinct context and signal depending on the situation (Keenleyside 1955; Losey and Sevenster 1995). Further analysis is required to fully understand the situational factors that influence the headstanding behavior, including potential triggers and contextual cues.

In summary, our study confirms that headstanding is a widespread behavior among juvenile freshwater fish of various species. Typically, a single fish initiates the behavior, which is often followed by others, resulting in multiple fish standing with their heads down. The distances between headstanding individuals vary depending on the transparency of the water, implying that this behavior is influenced, at least in part, by visual perception. We provide an overview of possible explanations for this behavior, suggesting that it may serve as a signal for complex social interactions or environmental awareness among freshwater fish, which warrant further exploration.

Future research should focus on elucidating the functional significance of headstanding, examining its prevalence across different life stages or environmental contexts, and its potential role in interspecies interactions. By integrating these behavioral observations with ecological and physiological studies, we can gain a more comprehensive understanding of the adaptive strategies employed by these species. We believe that exploring these questions has much to tell us not only about life beneath the water surface but also about the evolution of social groups and the individual benefits of cooperation with others.

## Author Contributions

**Michal Tušer:** conceptualization (equal), data curation (lead), formal analysis (lead), funding acquisition (lead), investigation (equal), methodology (equal), resources (lead), visualization (lead), writing – original draft (lead), writing – review and editing (lead). **Jaroslava Frouzová:** conceptualization (equal), investigation (equal), methodology (equal), writing – original draft (supporting), writing – review and editing (supporting).

## Conflicts of Interest

The authors declare no conflicts of interest.

## Supporting information


Appendix S1

## Data Availability

The data that support the findings of this study are available from the corresponding author upon request.
