# Supplementary material for: Enigmatic Headstands in European Freshwater Fish Species
Source: Ecol Evol. 2025 Feb 15;15(2):e71005. doi: 10.1002/ece3.71005 (PMC11829109; doi:10.1002/ece3.71005)
Supplement: Supplementary file 1 — Appendix S1 [file ECE3-15-e71005-s001.pdf]

Table S1. Phytoplankton concentrations in the Římov Reservoir, Czech Republic.

Sampling date	Chlorophyll α (ethanol) $\mu g \cdot L^{-1}$
2019-07-15	18.0
2019-08-05	13.9
2022-08-08	34.0
2022-08-29	63.1

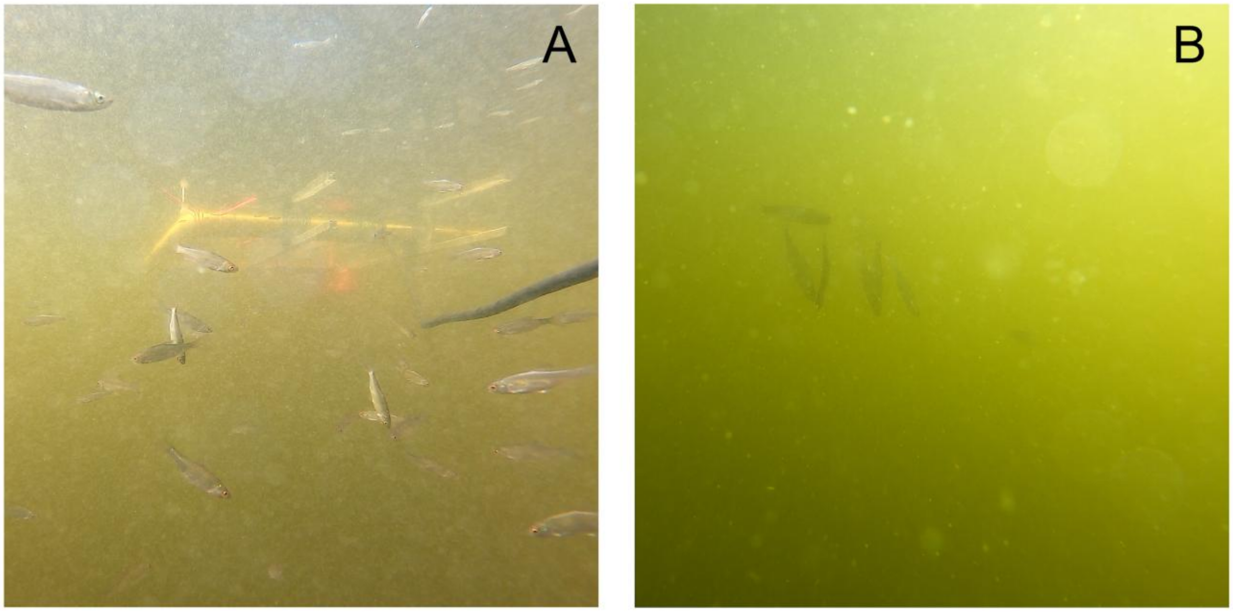

Figure S1. Water transparency conditions in summer and fall in 2019 (A) and 2022 (B) showed remarkable differences.

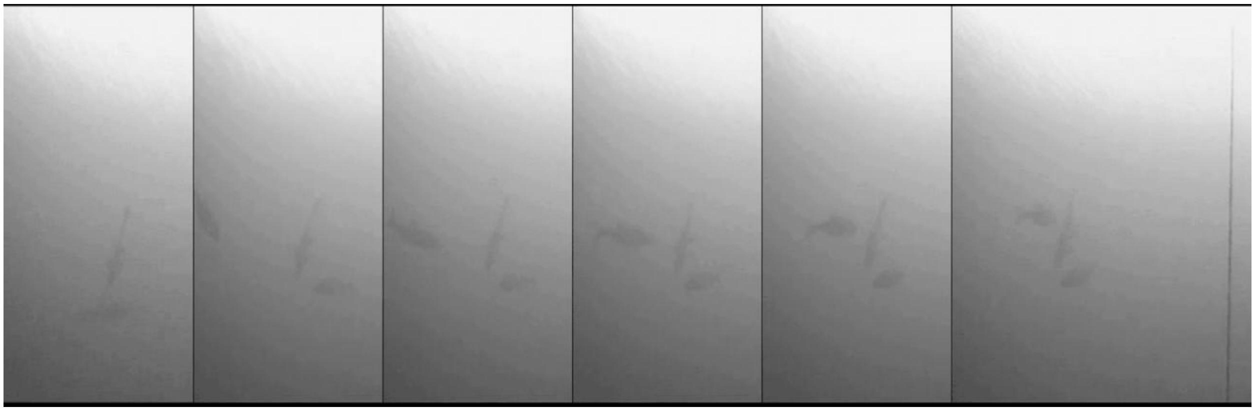

Figure S2. A video sequence of an adult roach (*Rutilus rutilus*) performing a headstand with two approaching shoal companions. The video was recorded in May 2005 in the Římov Reservoir, Czech Republic. The optical camera was pointed at the water surface at an angle of 45°.
